# Natural *Saccharomyces cerevisiae* Strain Reveals Peculiar Genomic Traits for Starch-to-Bioethanol Production: the Design of an Amylolytic Consolidated Bioprocessing Yeast

**DOI:** 10.3389/fmicb.2021.768562

**Published:** 2022-01-20

**Authors:** Nicoletta Gronchi, Nicola De Bernardini, Rosemary A. Cripwell, Laura Treu, Stefano Campanaro, Marina Basaglia, Maria R. Foulquié-Moreno, Johan M. Thevelein, Willem H. Van Zyl, Lorenzo Favaro, Sergio Casella

**Affiliations:** ^1^Department of Agronomy, Food, Natural Resources, Animals and Environment (DAFNAE), University of Padua, Legnaro, Italy; ^2^Department of Biology, University of Padua, Padua, Italy; ^3^Department of Microbiology, Stellenbosch University, Stellenbosch, South Africa; ^4^Department of Molecular Microbiology, VIB, KU Leuven, Leuven, Belgium; ^5^NovelYeast Bv, Open Bio-Incubator, Erasmus High School, Jette, Belgium

**Keywords:** *Saccharomyces cerevisiae*, delta integration, CRISPR/Cas9, starch, Ethanol Red, consolidated bioprocessing, amylases, bioethanol

## Abstract

Natural yeast with superior fermentative traits can serve as a platform for the development of recombinant strains that can be used to improve the sustainability of bioethanol production from starch. This process will benefit from a consolidated bioprocessing (CBP) approach where an engineered strain producing amylases directly converts starch into ethanol. The yeast *Saccharomyces cerevisiae* L20, previously selected as outperforming the benchmark yeast Ethanol Red, was here subjected to a comparative genomic investigation using a dataset of industrial *S. cerevisiae* strains. Along with Ethanol Red, strain L20 was then engineered for the expression of α-amylase *amyA* and glucoamylase *glaA* genes from *Aspergillus tubingensis* by employing two different approaches (delta integration and CRISPR/Cas9). A correlation between the number of integrated copies and the hydrolytic abilities of the recombinants was investigated. L20 demonstrated important traits for the construction of a proficient CBP yeast. Despite showing a close relatedness to commercial wine yeast and the benchmark Ethanol Red, a unique profile of gene copy number variations (CNVs) was found in L20, mainly encoding membrane transporters and secretion pathway proteins but also the fermentative metabolism. Moreover, the genome annotation disclosed seven open reading frames (ORFs) in L20 that are absent in the reference S288C genome. Genome engineering was successfully implemented for amylase production. However, with equal amylase gene copies, L20 proved its proficiency as a good enzyme secretor by exhibiting a markedly higher amylolytic activity than Ethanol Red, in compliance to the findings of the genomic exploration. The recombinant L20 dT8 exhibited the highest amylolytic activity and produced more than 4 g/L of ethanol from 2% starch in a CBP setting without the addition of supplementary enzymes. Based on the performance of this strain, an amylase/glucoamylase ratio of 1:2.5 was suggested as baseline for further improvement of the CBP ability. Overall, L20 showed important traits for the future construction of a proficient CBP yeast. As such, this work shows that natural *S. cerevisiae* strains can be used for the expression of foreign secreted enzymes, paving the way to strain improvement for the starch-to-bioethanol route.

## Introduction

The increasing global fuel demand claims for the develop of a sustainable and cost-effective technology to convert polysaccharides into bioethanol. Nowadays, the production of ethanol from starch is particularly significant in the United States and Europe, being the leading producers of first-generation bioethanol from corn and wheat, respectively. Being more readily degradable than lignocellulose, starch is the preferred raw material for conversion into ethanol. Therefore, it is not surprising that one-third of the current global corn production is dedicated to the biofuel industry (Mohanty and Swain, 2019). However, despite the benefits from the reduced petroleum reliance, the use of corn contributes to the price increase in food and feed commodities, as well as the depletion of water resources and soil degradation. Alternatively, the use of starchy residual biomass from forestry, agricultural and industrial activities has been proposed as second-generation feedstock to preserve the food supply chain and to reduce the environmental threat (Lin and Tanaka, 2006; Castro et al., 2011; Vohra et al., 2014; Gupta and Verma, 2015; Aditiya et al., 2016; Zabed et al., 2017; Robak and Balcerek, 2018; Myburgh et al., 2019).

The starch-to-ethanol conversion is a well-established and technically mature technology. The process involves significant heat-intensive steps for starch liquefaction, as well as the use of commercial thermostable hydrolase mixtures (α-amylase and glucoamylase) for the complete saccharification of the substrate (Ishizaki and Hasumi, 2014; Vohra et al., 2014; Cinelli et al., 2015; Zabed et al., 2017; Cripwell et al., 2020). With the aim of limiting the operational costs as well as the capital by employing waste biomass, the integration of all steps into a single fermentative unit simplifies the industrial process and is expected to save up to 10–50% of the cost (Lynd et al., 2008; Brown et al., 2020; Cunha et al., 2020). In this scenario the employment of a consolidated bioprocessing (CBP) yeast, able to simultaneously hydrolyze starchy biomass and directly ferment the resulting glucose at fermentation temperatures would represent a cost-savings approach. However, to date, no natural yeast isolate has been described to perform CBP for sustainable bioethanol production (Favaro et al., 2013, 2015; Kricka et al., 2014; Cripwell et al., 2019a; Adegboye et al., 2021).

The ethanologenic yeast *Saccharomyces cerevisiae* represents the ultimate candidate for bioethanol production due to the ease of cultivation and the generally recognized as safe (GRAS) and qualified presumption of safety (QPS) status (Sharma et al., 2018; Favaro et al., 2019b). Moreover, robust industrial strains have been adapted to stressful conditions and present favorable traits such as high fermentation rate, general robustness, tolerance to low pH and osmotic stress. The major limitation, however, is their inability to produce amylases (Görgens et al., 2015; Cripwell et al., 2020).

Despite the large employment of *S. cerevisiae* in biotechnological research, only CBP yeasts with limited amylolytic activity are currently employed on an industrial scale. The genome engineering for amylase expression in industrial *S. cerevisiae* strains has already been reported by integration of heterologous genes at delta sequences of the Ty retrotransposon (Cho et al., 1999; Kang et al., 2003; Favaro et al., 2010, 2015; Cripwell et al., 2019a) or ribosomal DNA (Lopes et al., 1989, 1996; Nieto et al., 1999; Choi et al., 2002; Liao et al., 2012). Although these strategies are known as very efficient in *S. cerevisiae* because of the native homologous recombination machinery, the inserts often result in long tandem repeats at one location leading to genome instability and unstable phenotypes. Likewise, multiple chromosome integrations can be hampered by the limited availability of selective markers.

Genetic modification of complex industrial yeast has advanced rapidly with the use of the Clustered Regularly Interspaced Short Palindromic Repeats (CRISPR)/CRISPR associated (Cas) protein system, which is by now widely considered as the technology of choice for metabolic engineering (Jansen et al., 2017; Zhang et al., 2020; Adegboye et al., 2021; Riley and Guss, 2021). Compared to other endonuclease-based and *in vivo* recombineering methods, it has proven to be a fast, marker-free, versatile, and most importantly site-directed genome-editing technique (Jakočiūnas et al., 2016; Roggenkamp et al., 2017).

From an industrial perspective, complete starch hydrolysis without liquefaction can only be achieved by a CBP yeast co-producing raw starch α-amylase and glucoamylase genes at high titers and able to ferment raw starch at high substrate loading (Cripwell et al., 2020; den Haan et al., 2021). Few groups have reported successful bioethanol production from raw corn starch using recombinant *S. cerevisiae* strains, mostly developed from laboratory backgrounds (Murai et al., 1998; Khaw et al., 2006; Yamada et al., 2009; Favaro et al., 2015; Cripwell et al., 2019a,b). Moreover, the exploration of the immense, and still largely unknown, potential of natural yeast strains could be of great relevance for the improvement of the starch-to-ethanol process (Favaro et al., 2019b). As reported in literature, natural yeast isolates have been screened for lignocellulosic bioethanol production (Basso et al., 2008; Jansen et al., 2017) but only a little is known for the starch process (Mohd Azhar et al., 2017; Favaro et al., 2019b; Cripwell et al., 2020). For instance, Gronchi et al. (2019) evaluated a cluster of natural *S. cerevisiae* isolates in simultaneous saccharification and fermentation (SSF) of raw starch and identified *S. cerevisiae* L20 as outperforming the industrial benchmark *S. cerevisiae* Ethanol Red (Lesaffre, France), which is one of the most widely used yeast strains for first-generation bioethanol production.

In this study, the L20 strain was examined at a genome level to highlight possible traits that could elucidate its superior fermentative abilities. The genome was assembled *de novo* by a hybrid Illumina/Nanopore approach to increase the genome completeness, and then subjected to a comparative analysis with a dataset of other *S. cerevisiae* strains. The dataset was constructed with the deposited genomic sequences of *S. cerevisiae* strains that are involved in alcoholic beverages and bioethanol production. The genome of the strain Ethanol Red was also sequenced and included in the dataset as a benchmark. Strain L20, which showed a unique amplification profile of genes, was then selected as suitable candidate for genome engineering in order to develop an efficient CBP strain. The α-amylase (*amyA*) and glucoamylase (*glaA*) genes from *Aspergillus tubingensis*, previously employed by Viktor et al. (2013) on a multicopy plasmid, were cloned and stably expressed in both strains L20 and Ethanol Red by adopting delta integration and CRISPR/Cas9 strategies. The recombinant strains were investigated with regards to their enzymatic and fermenting abilities on corn starch, giving particular attention to the correlation between the gene copy number and hydrolytic activity.

The results demonstrated that *S. cerevisiae* L20 exhibited a great potential for the application in the bioethanol industry. At the same time, the strain was successfully employed as microbial platform for the development of a starch-CBP yeast. For the first time, a natural yeast strain has been engineered by the CRISPR/Cas9 technology for fungal amylase production, representing the earliest example of a drop-in yeast for the starch-to-ethanol industry.

## Materials and Methods

### Media and Culture Conditions

All chemicals were of analytical grade and were obtained from Sigma-Aldrich, unless stated otherwise. *Escherichia coli* cells were grown at 37°C in Luria Bertani broth (LB; g/L: yeast extract, 5; tryptone, 10; NaCl, 10) supplemented with ampicillin (100 mg/L). *S. cerevisiae* strains were maintained in yeast extract peptone dextrose (YPD; g/L: yeast extract, 10; peptone, 20; glucose, 20) or on selective YPD plates supplemented with agar (15 g/L) and antibiotics: geneticin sulfate (200 mg/L, Sigma-Aldrich), hygromycin B (300 mg/L, Invivogen), or nourseothricin (100 mg/L, Jena Bioscience). Ampicillin and streptomycin (75 mg/L, Sigma-Aldrich) were added to prevent bacterial contamination during fermentation. Yeast nitrogen base plates (YNB; g/L: yeast nitrogen base, 6.7; agar, 15) containing 0.2% soluble corn starch were used for amylase plate assays.

### Strains and Plasmids

The strains and plasmids used in this study are summarized in Table 1.

**TABLE 1:** Strains and plasmids used in this study.

Strain and plasmids	Description	Source/References
*E. coli* NEB 5-alpha	*fhu*A2 Δ(*arg*F-*lac*Z)U169 *pho*A *gln*V44 Φ80 Δ(*lac*Z)M15 *gyr*A96 *rec*A1 *rel*A1 *end*A1 *thi*-1 *hsd*R17	New England Biolabs
*S. cerevisiae* strains		
Ethanol Red*	MATa/α prototroph, industrial strain	Fermentis, France
L20	Natural isolate from a winery with outstanding fermenting abilities	Gronchi et al., 2019
L20 dT8	δ-integration of *ENO1*_P_*-amyA-ENO1*_T_, *ENO1*_P_*-glaA-ENO1*_T_ and *TEF1*_P_*-kanMX-TEF1*_T_	This study
L20 dT12	δ-integration of *ENO1*_P_*-amyA-ENO1*_T_, *ENO1*_P_*-glaA-ENO1*_T_ and *TEF1*_P_*-kanMX-TEF1*_T_	This study
L20 dT25	δ-integration of *ENO1*_P_*-amyA-ENO1*_T_, *ENO1*_P_*-glaA-ENO1*_T_ and *TEF1*_P_*-kanMX-TEF1*_T_	This study
L20 dT53	δ-integration of *ENO1*_P_*-amyA-ENO1*_T_, *ENO1*_P_*-glaA-ENO1*_T_ and *TEF1*_P_*-kanMX-TEF1*_T_	This study
L20 IS4.1-A	CRISPR-based integration of *ENO1*_P_*-amyA-ENO1*_T_ at locus IS4.1	This study
L20 IS4.1-AG	CRISPR-based integration of *ENO1*_P_*-amyA-ENO1*_T_-*ENO1*_P_*-glaA-ENO1*_T_ at locus IS4.1	This study
L20 IS7.1-G	CRISPR-based integration of *ENO1*_P_*-glaA-ENO1*_T_ at locus IS7.1	This study
L20 IS7.1-GA	CRISPR-based integration of *ENO1*_P_*-glaA-ENO1*_T_-*ENO1*_P_*-amyA-ENO1*_T_ at locus IS7.1	This study
L20 IS4.1-A_IS7.1-G	CRISPR-based integration of *ENO1*_P_*-amyA-ENO1*_T_ at locus IS4.1 and *ENO1*_P_*-glaA-ENO1*_T_ at locus IS7.1	This study
L20 IS4.1-AG_IS7.1-GA	CRISPR-based integration of *ENO1*_P_*-amyA-ENO1*_T_-*ENO1*_P_*-glaA-ENO1*_T_ at locus IS4.1 and *ENO1*_P_*-glaA-ENO1*_T_-*ENO1*_P_*-amyA-ENO1*_T_ at locus IS7.1	This study
ER dT16	δ-integration of *ENO1*_P_*-amyA-ENO1*_T_, *ENO1*_P_*-glaA-ENO1*_T_ and *TEF1*_P_*-kanMX-TEF1*_T_	This study
ER dT17	δ-integration of *ENO1*_P_*-amyA-ENO1*_T_, *ENO1*_P_*-glaA-ENO1*_T_ and *TEF1*_P_*-kanMX-TEF1*_T_	This study
ER dT22	δ-integration of *ENO1*_P_*-amyA-ENO1*_T_, *ENO1*_P_*-glaA-ENO1*_T_ and *TEF1*_P_*-kanMX-TEF1*_T_	This study
ER IS4.1-A	CRISPR-based integration of *ENO1*_P_*-amyA-ENO1*_T_ at locus IS4.1	This study
ER IS4.1-AG	CRISPR-based integration of *ENO1*_P_*-amyA-ENO1*_T_-*ENO1*_P_*-glaA-ENO1*_T_ at locus IS4.1	This study
ER IS7.1-G	CRISPR-based integration of *ENO1*_P_*-glaA-ENO1*_T_ at locus IS7.1	This study
ER IS7.1-GA	CRISPR-based integration of *ENO1*_P_*-glaA-ENO1*_T_-*ENO1*_P_*-amyA-ENO1*_T_ at locus IS7.1	This study
Plasmids		
yBBH1-AmyA	*bla URA3 ENO1*_P_ *-amyA-ENO1*_T_	Viktor et al., 2013
yBBH1-GlaA	*bla URA3 ENO1*_P_ *-glaA-ENO1*_T_	Viktor et al., 2013
pBKD2	*amp*δ*-ENO1*_P_*-ENO1*_T_ *TEF1*_P_*-kanMX-TEF1*_T_-δ	McBride et al., 2008
pTEF-Cas9-kanMX	*TEF1*_P_-*Cas9*-CYC1_T_-T *TEF1*_P_*-kanMX-TEF1*_T_	Claes et al., 2020
p426-SNR52_P_-IS4.1.CAN1.Y-SUP4_T_	gRNA IS4.1-*TEF1*_P_*-cloNAT-TEF1*_T_	Claes et al., 2020
p426-SNR52_P_-IS7.1.CAN1.Y-SUP4_T_	gRNA IS7.1-*TEF1*_P_*-cloNAT-TEF1*_T_	Claes et al., 2020
p426-hph-IS4.1	*TEF1*_P_*-hph-TEF1*_T_, homologous regions for IS4.1 locus	Claes et al., 2020
p426-hph-IS4.1-A	*TEF1*_P_*-hph-TEF1*_T_ -*ENO1*_P_*-amyA-ENO1*_T_	This study
p426-hph-IS4.1-AG	*TEF1*_P_*-hph-TEF1*_T_-*ENO1*_P_*-amyA-ENO1*_T_*-ENO1*_P_*-glaA-ENO1*_T_	This study
p426-hph-IS7.1	*TEF1*_P_*-hph-TEF1*_T_, homologous regions for IS7.1 locus	Claes et al., 2020
p426-hph-IS7.1-G	*TEF1*_P_*-hph-TEF1*_T_- *ENO1*_P_*-glaA-ENO1*_T_	This study
p426-hph-IS7.1-GA	*TEF1*_P_*-hph-TEF1*_T_-*ENO1*_P_*-glaA-ENO1*_T_-*ENO1*_P_*-amyA-ENO1*_T_	This study

*Version 1.

### Whole-Genome Sequencing

Genomic DNA from *S. cerevisiae* L20 and Ethanol Red strains was isolated from overnight cultures using zymolyase digestion and standard phenol-chloroform extraction (Treu et al., 2014). A combined sequencing approach was then applied using Illumina and Oxford Nanopore MinION single-molecule sequencing. The Illumina library was generated using the TruSeq DNA PCR-Free Library Prep Kit (Illumina Inc., San Diego, CA, United States) and Covaris S2 (Woburn, MA, United States) for a 550-bp average fragment size. The library was loaded onto the flow cell provided in the NextSeq 500 Reagent kit v2 (150 cycles, Illumina Inc., San Diego, CA, United States) and sequenced on a NextSeq 500 (Illumina Inc., San Diego, CA, United States) platform with a paired-end protocol and read lengths of 151 bp at the CRIBI Biotechnology Center (Padua, Italy). The Nanopore library was prepared according to the SQK-LSK109/SQK-RBK004 ligation sequencing kit and sequenced on a FLO-MIN106 R9/FLO-MIN106 D flowcell; a detailed procedure of DNA extraction/purification and library preparation was reported in Basile et al. (2021). The genome assembly was performed with a *de novo* approach with an in-house pipeline developed to combine Nanopore and Illumina sequences analysis. Briefly, the long reads were corrected and assembled with the Canu (Koren et al., 2017) software. The obtained contigs were polished with Pilon (Walker et al., 2014) software using the independent high-quality Illumina sequences. A whole-genome alignment was then obtained using Mauve software (Darling et al., 2010) to highlight genome completeness and structural variants in comparison to the reference *S. cerevisiae* S288C. For the CRISPR/Cas9 application, the non-coding loci IS4.1 and IS7.1 were selected from Claes et al. (2020) and confirmed as suitable targets for p426-SNR52_P_-IS4.1.CAN1.Y-SUP4_T_ and p426-SNR52_P_-IS7.1.CAN1.Y-SUP4_T_ guide RNA vectors in L20 and Ethanol Red strains.

The genomic DNA was isolated from the recombinant strains for Illumina sequencing to verify the copy number of integrated amylase cassettes. DNA was extracted from overnight cultures according to DNeasy PowerSoil Kit (Qiagen). An additional cleaning step with phenol:chloroform:isoamyl alcohol (PCI; 25:24:1, v/v) solution (Sigma-Aldrich) was performed before DNA isolation. Illumina library and assembly were performed as previously described for host strains. The sequences of integrated genes (*amyA* and *glaA*) and single-copy reference genes (*ACT1*, *ALG9*, *PGK1*, and *TFC1*) were used as queries for BLAST analyses. The integrated gene copy numbers were assessed based on the ratio between the average coverage of selected reference genes and the average coverage of the heterologous genes (Cripwell et al., 2019a).

### Comparative Genome Investigation

A comparative genomic approach was used to characterize L20. Fifty-four *S. cerevisiae* strains, whose genome has been previously deposited in online repositories, were selected due to their commercial/industrial relevance. Wine and beer-producing strains were chosen because of the L20’s enological background, and bioethanol producing strains were included in view of the final application (CBP strain development). The S288C and Ethanol Red genomes were also included. The selected strains were grouped according to their industrial application (Table 2).

**TABLE 2:** Yeast strains used in the comparative genomic analysis.

Strain name	Origin/Application	Accession number	References
S288C	Laboratory reference strain	SRR2968033	*Saccharomyces* Genome Database
L20	Natural vineyard isolate	This study	Gronchi et al., 2019
***Bioethanol***			
Ethanol Red	Industrial bioethanol production from corn (Lessafre)	This study	Gronchi et al., 2019
ISO12	Haploid spore of Ethanol Red	SRR2002960	Wallace-Salinas et al., 2015
Y22-3	Industrial bioethanol production from lignocellulose	SRR2989884	McIlwain et al., 2016
BG1	Industrial bioethanol production from sugarcane	SRR403237	Dunn et al., 2012
CAT-1	Industrial bioethanol production from sugarcane	SRR5678610	Babrzadeh et al., 2012
SA-1	Industrial bioethanol production from sugarcane	SRR8455574	Nagamatsu et al., 2021
VR-1	Industrial bioethanol production from sugarcane	SRR5678581	Gallone et al., 2016
CBS7959	Industrial bioethanol production from sugarcane	ERR1309137	Peter et al., 2018
CBS7960	Industrial bioethanol production from sugarcane	ERR1308983	Peter et al., 2018
CBS7961	Industrial bioethanol production from sugarcane	ERR1309524	Peter et al., 2018
CBS7962	Industrial bioethanol production from sugarcane	ERR1308731	Peter et al., 2018
CBS7963	Industrial bioethanol production from sugarcane	ERR1309041	Peter et al., 2018
CBS7964	Industrial bioethanol production from sugarcane	ERR1308847	Peter et al., 2018
M1.1	Industrial bioethanol production from sugarcane	ERR1308918	Antonangelo et al., 2013
M.9.1	Industrial bioethanol production from sugarcane	ERR1308583	Antonangelo et al., 2013
M.14.1	Industrial bioethanol production from sugarcane	ERR1309375	Antonangelo et al., 2013
RP.10.4	Industrial bioethanol production from sugarcane	ERR1309497	Antonangelo et al., 2013
RP.10.13	Industrial bioethanol production from sugarcane	ERR1309103	Antonangelo et al., 2013
RP.10.14	Industrial bioethanol production from sugarcane	ERR1308589	Antonangelo et al., 2013
RP11.4.1	Industrial bioethanol production from sugarcane	ERR1309463	Antonangelo et al., 2013
RP11.4.11	Industrial bioethanol production from sugarcane	ERR1309424	Antonangelo et al., 2013
RP11.4.14	Industrial bioethanol production from sugarcane	ERR1309157	Antonangelo et al., 2013
SA.1.5	Industrial bioethanol production from sugarcane	ERR1309329	Antonangelo et al., 2013
SA.9.2.BL3	Industrial bioethanol production from sugarcane	ERR1309339	Antonangelo et al., 2013
SA.9.3.VR1	Industrial bioethanol production from sugarcane	ERR1308862	Antonangelo et al., 2013
SA.9.4.BR2	Industrial bioethanol production from sugarcane	ERR1309465	Antonangelo et al., 2013
SA.9.4.VL4	Industrial bioethanol production from sugarcane	ERR1309264	Antonangelo et al., 2013
SA.10.1.VL1	Industrial bioethanol production from sugarcane	ERR1309357, ERR1309110	Antonangelo et al., 2013
SA.10.1.VR4	Industrial bioethanol production from sugarcane	ERR1309211	Antonangelo et al., 2013
SM.8.2.C13	Industrial bioethanol production from sugarcane	ERR1309287	Antonangelo et al., 2013
SM.8.7.BR1	Industrial bioethanol production from sugarcane	ERR1308859	Antonangelo et al., 2013
SM.8.7.L8	Industrial bioethanol production from sugarcane	ERR1309346	Antonangelo et al., 2013
SM.8.7.L9	Industrial bioethanol production from sugarcane	ERR1308869	Antonangelo et al., 2013
SM.8.8.BL1	Industrial bioethanol production from sugarcane	ERR1308925	Antonangelo et al., 2013
SM.8.8.CVR1	Industrial bioethanol production from sugarcane	ERR1308810	Antonangelo et al., 2013
SM.9.1.AL1	Industrial bioethanol production from sugarcane	ERR1309387	Antonangelo et al., 2013
SM.9.1.BL7	Industrial bioethanol production from sugarcane	ERR1309294	Antonangelo et al., 2013
SM.9.2.BR3(L)	Industrial bioethanol production from sugarcane	ERR1308860	Antonangelo et al., 2013
SM.9.4.BL2	Industrial bioethanol production from sugarcane	ERR1309184	Antonangelo et al., 2013
SM.9.4.BR1	Industrial bioethanol production from sugarcane	ERR1309123	Antonangelo et al., 2013
SM.9.4.BR2	Industrial bioethanol production from sugarcane	ERR1309134	Antonangelo et al., 2013
VF8 6	Industrial bioethanol production from sugarcane	ERR1309220	Antonangelo et al., 2013
***Wine***			
BM45	Lallemand	ERR756199	Legras et al., 2018
ICVD254	Lallemand	ERR756200	Legras et al., 2018
JCY254	Lallemand	ERR756208	Legras et al., 2018
QA23	Lallemand	ERR756198	Legras et al., 2018
***Bioethanol***			
AWRI796	Maurivin	SRR2967949	Borneman et al., 2016
WLP705	White Labs	SRR2968044	Borneman et al., 2016
EC1118	Lallemand	SRR2967901	Borneman et al., 2016
VL1	Zymaflore	ERR756227	Legras et al., 2018
***Ale/Rhum***			
CLIB382	Beer production	SRR6114133	Schacherer et al., 2009
L328	Cachaca production	ERR756202	Legras et al., 2018
WLP800	White Labs	SRR2968034	Borneman et al., 2016
WLP001	White Labs	SRR2968036	Borneman et al., 2016
WY1084	Wyeast	SRR2968040	Borneman et al., 2016

*The genomes of S. cerevisiae L20 and Ethanol Red were sequenced and assembled de novo in this study. The accession numbers (European Nucleotide Archive/Sequence Read Archive) for raw sequencing data are reported for other strains.*

#### Variant Calling and Phylogenetic Analysis

The genetic variants present in open reading frames (ORFs) were investigated for their potential as a source of phenotypic variation. Variant calling analysis was performed following the Genome Analysis Toolkit (GATK4, v4.1.9.0) Best Practices as first discussed by DePristo et al. (2011). Through the comparison with a reference genome, this framework allows the discovery of small genetic variants, such as single nucleotide polymorphisms (SNPs) and insertion-deletions (INDELs), and the genotyping of multiple samples simultaneously. The strain selected for all the reference-based analyses was the *S. cerevisiae* S288C R64-1-1. The key phases of the pipeline were applied as previously described by Basile et al. (2021). Briefly, (I) filtered reads were aligned using bwa mem (v0.7.17); (II) base quality scores were recalibrated using a machine learning model implemented in “BQSR”; (III) variants were identified using the haplotype caller algorithm; (IV) detected variants were filtered using a variant quality score recalibration model trained on three different subsets of a previously published dataset (Peter et al., 2018). Functional effect prediction and genetic variants annotation were performed with the SnpEff software (v5.0) (Cingolani et al., 2012).

The genetic variants of the yeast dataset were identified and only the biallelic SNPs were retained using VCFtools (v0.1.16) for the phylogenetic analysis. Overall, the resulting subset contains 299,604 strain-specific variants which have been subsequently processed to generate a multiple sequence alignment (MSA) in FASTA format required as input by IQ-TREE v2.0.3 (Nguyen et al., 2015). The tools used for the conversion include “vcf-to-tab” from the VCFtools suite (Danecek et al., 2011), GNU Datamash (Free Software Foundation Inc., 2014) and a custom Perl (Wall et al., 2000) script. Finally, IQ-TREE was used to reconstruct a maximum likelihood (ML) phylogenetic tree. The substitution model (SYM + R3) adopted was selected with ModelFinder (Kalyaanamoorthy et al., 2017) and the robustness of the topology was further assessed using 500 ultrafast bootstrap iterations (Hoang et al., 2018). The Interactive Tree Of Life (iTOL)^1^ was finally used for the graphic representation of the phylogenetic tree (Letunic and Bork, 2019).

#### *Saccharomyces cerevisiae* L20 Open Reading Frame Detection and Copy Number Analysis

The similarity between L20 and S288C orthologous genes was estimated using two strategies: (1) annotated genes from the reference were mapped to the target assembly using Liftoff (v1.6.1) to predict which are common; differences among strains were determined (2) by predicting the total ORFs and extracting L20 specific genes and (3) by predicting ORFs in L20 accessory region with respect to the reference. To limit the gene finding process only to strain-specific regions of L20, a strategy previously reported by Basile et al. (2021) was implemented. It consisted in the identification of strain-specific regions of at least 500 bp with AGEnt (Ozer et al., 2014) followed by the prediction of protein-encoding genes within these regions. GeneMark-ES (v4.67) was used in both analyses, accounting for fungal-specific intron organization and assuming a maximum intron length of 500 for the prediction (Ter-Hovhannisyan et al., 2008). Afterward, the genes identified in L20 were translated into protein sequence and clustered with the reference proteome using cd-hit software (v4.8.1) (Fu et al., 2012). The thresholds used for clustering were minimum length of 100 nucleotides per sequence and minimum overlap with the cluster longest sequence of at least 10%. Different identity thresholds between clustered sequences have been tested, ranging from 80 to 95%, but only results derived from the most conservative ones (80%) were used for further analysis. The ORFs found in L20 but not in the reference S288C were annotated using RPS-BLAST (v2.6.0+).

The copy number variations (CNVs) were estimated based on whole genome sequencing data using CNVpytor (v1.0; Suvakov et al., 2021). The raw reads were polished using Trimmomatic (v.0.39), aligned to the reference genome with bwa (v.0.7.17) and eventually run with CNVpytor. The software estimated CNV values of entire genome regions based on read depth (RD) and allowed the extraction of predicted copy numbers. A deeper insight was dedicated to the CNV for the Gene Ontology (GO) terms “transmembrane transport” (GO:0090662; GO:0006899), “energy derivation by oxidation of organic compounds” (GO:0015980) and “response to stress” (GO:0006950) as annotated in AmiGO database for *S. cerevisiae* S288C and SGD database.

### DNA Manipulation and Yeast Transformation

Standard protocols were followed for DNA manipulations and *E. coli* transformation (Sambrook and Russell, 2001). Restriction enzymes were supplied by New England Biolabs or Thermofisher and used as recommended by the supplier. DNA was eluted from 1% agarose gels using the Wizard SV Gel and PCR Clean-Up System (Promega). Plasmids were isolated using the NucleoSpin Plasmid Easy Pure kit (Macherey-Nagel). The Q5 High Fidelity (New England Biolabs) polymerase was used for PCR amplification.

#### Delta Integration Approach

For the delta integration approach, linear donor DNA fragments were constructed by providing 500 bp-homologous flanking regions to the amylase cassettes. Briefly, the yBBH1-AmyA and yBBH1-GlaA plasmids (Viktor et al., 2013) were used as templates to amplify the δ-*ENO1*_P_-*amyA*-*ENO1*_T_ and δ-*ENO1*_P_-*glaA*-*ENO1*_T_ amylase fragments, respectively, using the ENO1_P_ Delta-L and ENO1_T_-R primers (Table 3). The geneticin marker was used for the selection: the *TEF1*_P_-*kanMX*-*TEF1*_T_-δ cassette was amplified from pBKD2 using ENO1_T_ marker-L and Delta-R Primers (Table 3) and ligated *in vivo* at the 3′ of the amylase fragments to generate δ-*ENO1*_P_-*amyA*-*ENO1*_T_-*kanMX*-δ and δ-*ENO1*_P_-*glaA*-*ENO1*_T_-*kanMX*-δ, respectively.

**TABLE 3:** Primers used for amplification of amylase cassettes, with italicized oligos representing regions for homologous recombination.

Primer name	Sequence (5′-3′)
**For delta integration**	
ENO1_P_ Delta-L	*TATACCTAATATTATAGCCTTTATCAACAATGGAATCCCAACAATTA TCTAATTACCCACATATATCTC*AACTAGTCTTCTAGGCGGGTT
ENO1_T_-R	GTCGAACAACGTTCTATTAGGAATGGCGGA
ENO1_T_ marker-L	CCTCCTAATGTGTCAATGATCATATTCTTA
Delta-R	*ATATTACGATTATTCCTCATTCCGTTTTAT*

**For Gibson Assembly**	

C-8465	*TCAGAAGCTTATCGATACCG*TACTGATCCGAGCTTCCACT
C-8466	*AAAGCGACACGTCGTGTCGAGGTACC*GTCGAACAACGTTCTATTAGG
C-8471	*TAATAGAACGTTGTTCGACG*TACTGATCCGAGCTTCCACT
C-8472	*TGCATGGGAGTCGAGGATC*GTCGAACAACGTTCTATTAGG
C-8467	*GCTAAAGCTTATCGATACCG*TACTGATCCGAGCTTCCACT
C-8468	*GCATCGTGCATGGGAGTCGAGGATCC*GTCGAACAACGTTCTATTAGG
C-8469	*AGAACGTTGTTCGACGGTAC*TACTGATCCGAGCTTCCACT
C-8470	*AGCGACACGTCGT*GTCGAGGTCGAACAACGTTCTATTAGG

*The respective restriction sites are underlined (BamHI = GGATCC; KpnI = GGTACC).*

Yeast cells were transformed according to Favaro et al. (2012) with δ-*ENO1*_P_-*amyA*-*ENO1*_T_, δ-*ENO1*_P_-*glaA*-*ENO1*_T_ and *TEF1*_P_-*kanMX*-*TEF1*_T_-δ cassettes simultaneously. In 0.2 cm electroporation cuvettes, an electric pulse of 1.4 kV, 200 Ω and 25 μF was applied using a Bio-Rad system (GenePluserXcell, Bio-Rad, Hercules, CA, United States). Cells were immediately suspended in 1 mL of YPD containing 1 M sorbitol (YPDS) and incubated at 30°C for 3 h to allow recovery. Electroporated cells were then spread onto YPD plates supplemented with geneticin and incubated at 30°C for 48 h. The recombinants were named according to the transformation method (“d” for delta integration and then consecutively numbered).

#### Clustered Regularly Interspaced Short Palindromic Repeat/CRISPR Associated Protein 9 Approach

**FIGURE 1:**
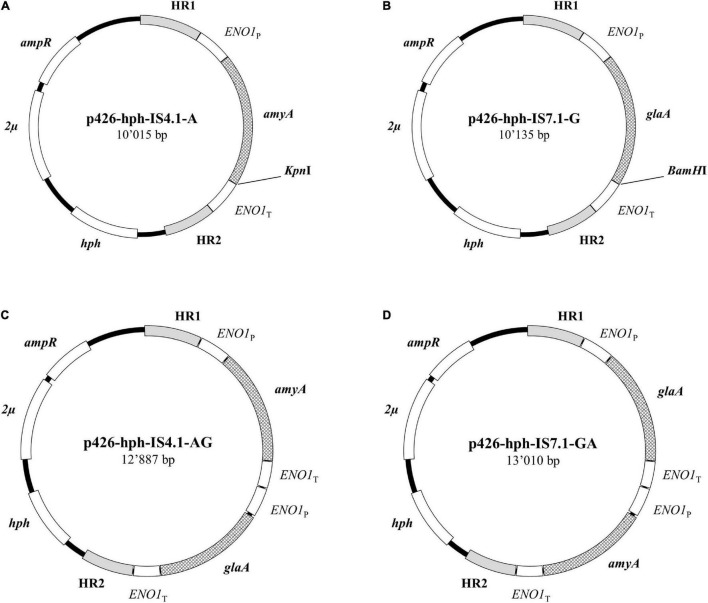
Donor DNA plasmids for the CRISPR/Cas9 method used in this study: plasmids were constructed to carry single *A. tubingensis amyA* **(A)** or *glaA* **(B)** or double cassette for the simultaneous expression of the *A. tubingensis amyA* and *glaA* genes **(C,D)**. IS4.1 and IS7.1 indicate the locus position for gene integration. The homologous regions (HR1 and HR2) are sequences flanking the designated genomic locus. All plasmids contained bacterial *ori* and *amp* genes for plasmid replication and ampicillin resistance, respectively.

A three plasmid-based approach was used for CRISPR/Cas9. The p426-hph vector was used as donor plasmid containing homologous regions for IS4.1 or IS7.1 loci and constructed to encode a single or a combination of *amyA* and *glaA* sequences (Table 1). The plasmid maps used in this study are reported in Figure 1. Briefly, the *ENO1*_P_-*amyA*-*ENO1*_T_ and *ENO1*_P_-*glaA*-*ENO1*_T_ cassettes were amplified from yBBH1 (Table 1) with primers reported in Table 3. The primers design and fragment assembly were performed according to the Gibson Assembly Cloning Kit (New England Biolabs) manufacturer’s recommendations. The plasmids were sent for Sanger sequencing (Mix2Seq; Eurofins Genomics, Germany).

The transformation was carried out using the LiAC/SS carrier DNA/PEG method (Gietz and Schiestl, 2007). Salmon sperm DNA (ssDNA 10 mg/mL) was purchased from Roche. The pTEF-Cas9-kanMX, p426-hph donor DNA and the gRNA plasmids were transformed separately in this order. Yeast cells were engineered for the expression of a single gene (donor vectors p426-hph-IS4.1-A for *amyA* in locus IS4.1; p426-hph-IS7.1-G for *glaA* in locus IS7.1), or double construct (p426-hph-IS4.1-AG and p426-hph-IS7.1-GA for *amyA* and *glaA* in locus IS4.1 and IS7.1, respectively; Table 1 and Figure 1). The L20 recombinants already transformed at IS4.1 and IS7.1 loci were subjected to a second round of transformation to target the alternative locus with a single or double cassette donor plasmid (Table 1 and Figure 1).

Cells were spread onto selective plates and incubated at 30°C for 48–72 h. The recombinants were named according to the donor plasmid/plasmids used (L20 IS4.1-A, IS4.1-AG, IS7.1-G, IS7.1-GA, IS4.1-A_IS7.1-G, IS4.1-AG_IS7.1-GA, Ethanol Red IS4.1-A, IS4.1-AG, IS7.1-G and IS7.1-GA).

### Confirmation and Characterization of Recombinants

#### Plate Assays and Plasmid Curing

Starch plate assays were used for qualitative analysis to verify the hydrolytic activity of the transformants. After 72 h growth in YPD, cultures were spotted onto YNB containing soluble corn starch and incubated for 48 h at 30°C. Strains expressing the amylase genes produced a clear surrounding halo after Lugol staining (Favaro et al., 2015). The yeast strains constructed using the CRISPR/CAS approach were subjected to sequential batch cultures using non-selective YPD broth for plasmid curing. The mitotic stability was verified according to Favaro et al. (2012). Single cell colonies were isolated on YPD plates by Singer Instruments MSM-400 micromanipulator.

#### Polymerase Chain Reaction Confirmation

Yeast colonies that produced clearing zones during plate assays were screened using the polymerase chain reaction (PCR) to confirm the presence of the integrated gene(s). Genomic DNA was extracted using the PCI solution with subsequent ethanol precipitation. PCR was performed with primers reported in Table 4. The genomic DNA of the parental strains was used as negative control.

**TABLE 4:** Primers used to confirm the integration of heterologous *amyA* and *glaA* genes in *S. cerevisiae*.

Primer	Binding site	Sequence
DeltaAmyA	Delta site Fw	GCATCAGCAACCTCTACAACA
DeltaGlaA	Delta site Fw	CATCCACACCTTTGATCCTG
C-2827	Chr IV Fw	CTCGTTGGTTGCAGTATACT
C-4330	Chr VII Fw	GGAGCAGACATCACTAAACG
C-8799	*amyA* Rv	CGCGTTTGTGGTGGCTATCCAGG
C-8797	*glaA* Rv	CGAGCAGAAAGCTCGTCGCCAT

*Primers were designed based on amyA and glaA sequences and used in combination to those specific for genomic flanking region.*

#### Protein Analysis

The supernatant from yeast cultures, grown for 24, 48, and 72 h, was denatured at 100°C for 3 min. The protein fractions were separated by SDS-PAGE using an 8% separation gel (Laemmli, 1970). Electrophoresis was carried out at 100 V for 90 min at room temperature and the proteins were visualized using the silver staining method (O’Connell and Stults, 1997). Supernatant from the parental strains was used as negative control. The broad-range PageRuler Prestained Protein Ladder (Fermentas) was used as a molecular mass marker.

#### Amylolytic Activity Assay

Recombinant strains were cultured in 20 mL YPD in 125 mL Erlenmeyer flasks with agitation at 120 rpm, with an initial optical density of 0.2 (OD_600_). The supernatant, collected after 24, 48, and 72 h of cultivation, was used to assess the enzymatic activity as described by Viktor et al. (2013). The total amylase activity was colorimetrically determined by using the DNS (3,5-dinitro salicylic acid) method described by Miller (1959) at 50°C for 5 min. For glucoamylase activity, 50 μL supernatant was incubated for 15 min with 450 μL of a 0.2% soluble corn starch solution (50°C, pH 5). The resulting glucose concentration was determined with the D-Glucose HK Assay Kit (Megazyme, Ireland) (adapted from Viktor et al., 2013). Enzymatic activities were expressed as nanokatals per mL (nKat/mL), which is defined as the enzyme activity needed to release 1 nmol of glucose per second per mL of culture. All experiments were carried out in triplicate. The parental strains were used as negative controls.

#### Consolidated Bioprocessing Fermentation Studies

Small-scale fermentations were performed on both soluble and raw corn starch in oxygen-limited conditions. Yeasts were cultured in 300 mL of YPD in 1-L Erlenmeyer flasks and incubated overnight at 30°C on a rotatory shaker at 120 rpm. Cells were collected by centrifugation for 5 min at 4000 rpm and inoculated at an OD_600_ value of 5 in 120-mL serum bottles containing 100 mL YP supplemented with 0.05% glucose, 2% soluble and raw starch and antibiotics (ampicillin and streptomycin) to prevent bacterial contamination. Serum bottles were sealed with rubber stoppers and provided with a needle for CO_2_ removal, then incubated at 30°C on a magnetic stirrer (Cimarec i Poly 15 Multipoint stirrer, Thermo Scientific) with agitation at 700 rpm. Samples were taken every 24 h, filtered through 0.22-μm for high-performance liquid chromatography (HPLC) analysis. The experiments were carried out in triplicate. Parental strains were used as negative control.

#### High-Performance Liquid Chromatography Analysis

Samples were analyzed for glucose, glycerol and ethanol through liquid chromatography using a Shimadzu Nexera HPLC system, equipped with a RID-10A refractive index detector (Shimadzu, Kyoto, Japan). The chromatographic separations were performed using a Rezex ROA-Organic Acid H^+^ (8%) column (300 mm 7.8 mm, Phenomenex, Torrance, CA, United States). The column temperature was set at 60°C and the analysis was performed at a flow rate of 0.6 mL/min using isocratic elution, with 2.5 mM H_2_SO_4_ as a mobile phase (Cagnin et al., 2019).

## Results and Discussion

From an industrial perspective, the implementation of a CBP yeast for complete starch utilization would require co-production of raw starch α-amylase and glucoamylase enzymes and fermentation at high substrate loadings. Considering the future development of an efficient CBP yeast, the host strain must, among other traits, yield superior ethanol levels. Bearing this in mind, *S. cerevisiae* L20, which was previously selected as a superior yeast strain under high gravity SSF conditions, was engineered to produce amylases. The ethanol yield of strain L20 was much greater than those exhibited by the industrial benchmark Ethanol Red. Genome sequencing data was used to unravel the basis of L20’s superior fermenting abilities and an engineering approach was pursued for the co-secretion of the *A. tubingensis* α-amylase *amyA* and glucoamylase *glaA*.

### *De novo* Genome Assemblies

The whole-genome sequence of L20 was obtained using a novel strategy that integrates MinION and Illumina technologies: the first platform is expected to produce robust scaffolds against which the Illumina reads can be mapped to in order to increase the assembly quality. The number of paired-end reads (2 × 150 bp) was 1,022,547, resulting in a 25-fold genome coverage. The number of MinION sequences was 58,954 with an average length of 6,649 bp. The *de novo* assembly generated a genome of 11.9 Mb, composed of 18 contigs with a N_50_ of 788,913 and 14 chromosomes assembled in a single contig. The genome size is comparable to the average of other natural and industrial *S. cerevisiae* strains (Gallone et al., 2016; Duan et al., 2018).

The whole-genome sequencing of Ethanol Red resulted in 136-fold genome coverage, with a total number of paired-end reads (2 × 150 bp) of 5,302,549. The number of MinION sequences was 121,382 with an average length of 4,493 bp. The *de novo* assembly produced a genome of 12.1 Mb, composed of 29 contigs with a N_50_ of 779,629.

Genome assembly details for all strains considered in this study are reported in Supplementary Table 1. Raw reads of *S. cerevisiae* L20 and Ethanol Red were deposited at GenBank under the BioProject accession number PRJNA762028.

### Comparative Genomic Investigation

The analysis of variants among the *S. cerevisiae* L20 and selected industrially relevant *S. cerevisiae* strains revealed 363,159 single nucleotide variants (SNV) in 343,963 loci. Variants were equally distributed among the 16 chromosomes, with an average rate of 1 every 33 bases. When grouped by type, 317,682 were SNPs (87.5%), and 24,668 (6.8%) and 20,809 (5.8%) were classified as insertion or deletion, respectively. A total of about 2.5 million effects were predicted, out of which 91.6% were found in non-coding regions. The 8.5% (212,137) and 6% (148,602) of effects were found in exons and intergenic regions, respectively. However, the majority of effects was detected within 5 kb upstream (5′-) or downstream (3′-) regions (44 and 42%, respectively). The details are reported in Supplementary Tables 1, 2.

#### Phylogenetic Analysis of Industrial Strains

**FIGURE 2:**
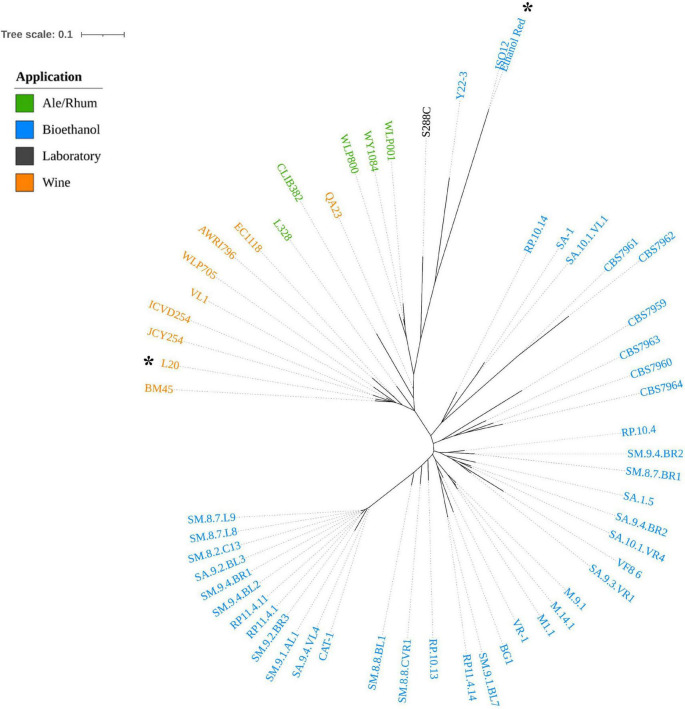
A maximum-likelihood phylogenetic tree based on SNP dataset representing the genetic distances among the 56 *S. cerevisiae* strains. The L20 and Ethanol Red strains sequenced in this study are marked with a black asterisk. The colors depict the industrial application of each strain (orange: Wine; green: Ale/Rhum; black: Laboratory; blue: Bioethanol).

The phylogenetic analysis was inferred on the dataset of small biallelic variants (299,604) and a maximum-likelihood tree was constructed and is shown in Figure 2.

The tree showed a clear separation of strains into two clusters: the first, represented by spirits-producing strains (Ale/Rhum and Wine), and the second including the fuel ethanol producers (Bioethanol). L20, which appeared to be clearly distinguished from other enological strains, was predicted as functionally related to commercial wine producers, in particular those isolated in Italy (BM45) and in France (JCY254 and ICVD254).

Interestingly, Ethanol Red, the benchmark yeast for first-generation bioethanol production, was phylogenetically assigned to the first cluster and closely related to the reference S288C. Ethanol Red and Y22-3 are the only bioethanol strains in this cluster. Y22-3 is a monospore engineered derivative of the stress-tolerant NRRL YB-210, which is a natural isolate from Costa Rican bananas and a progenitor of S288C (Mortimer and Johnston, 1986). However, the close relatedness to wine strains shows that Ethanol Red could share more genetic traits with domesticated strains rather than with the second cluster. These results are consistent with what was reported by Nagamatsu et al. (2021). However, Ethanol Red is still the most closely related strain to the sister branch of sugar-cane bioethanol strains, possibly representing a link between the two clusters. With the aim of selecting an alternative *S. cerevisiae* strain for the sustainable production of bioethanol from starch, these findings may indicate that enological yeast could be employed as promising host in genome engineering for the construction of a CBP starch-fermenting yeast.

#### Genomic Structural Analysis

**FIGURE 3:**
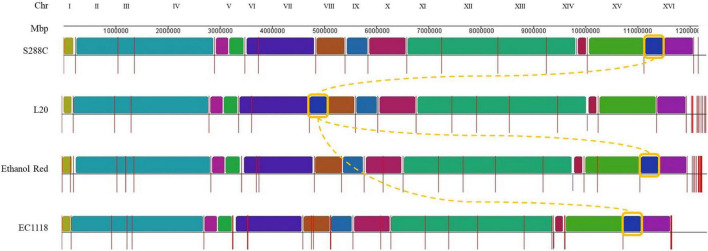
Multiple genome alignment of selected *S. cerevisiae* strains. The newly sequenced L20 and Ethanol Red genomes are compared to the reference S288C and with EC1118 strains. Chromosomes are ordered according to the S288C strain (first row) and syntenic regions are represented using different colors. Contiguous regions (chromosomes or scaffolds) are separated by red vertical bars. The translocation identified in L20 is highlighted using a yellow box.

The genome of L20 was assembled resulting in high-quality telomere-to-telomere reconstruction. The hybrid genomes obtained were used for the structural analysis by whole-genome alignment. The reference *S. cerevisiae* S288C R64-1-1 strain and one of the most studied industrial wine strains, EC1118 strain, were included (Figure 3).

Most of the L20 chromosomes assembled entirely in one contig except for chromosomes XII and XIV, which assembled in two fragments. The alignments in Figure 3 highlights a significant translocation between chromosomes VIII and XVI, which is a widespread translocation among enological yeast strains, although it has been identified in non-wine strains in the past as well (Perez-Ortin et al., 2002; Hou et al., 2014; Treu et al., 2014; García-Ríos and Guillamón, 2019; Crosato et al., 2020; Basile et al., 2021). It was correlated to an increased sulfur dioxide resistance, which is a critical parameter in winemaking. Since L20 was isolated from grape marcs (Gronchi et al., 2019), it can be assumed that such ecological background selected for this modification in L20 as well. This was further supported by the phylogenetic analysis (Figure 2) showing L20’ closest relatedness to commercial wine strains.

#### Exploration of Copy Number Variation

Yeast employed in industrial bioethanol fermentations are exposed to multiple stresses such as high sugar and ethanol concentrations, but also the presence of salts, sulfites, low pH, and bacterial contamination (Bauer and Pretorius, 2000; Favaro et al., 2019b; Brown et al., 2020). The variations of the gene copy number is usually associated with the adaptation to such specific conditions. Nagamatsu et al. (2021) examined the gene families having a positive selection in bioethanol yeast: due to the high metal concentration in sugarcane hydrolyzates, genes related to the metal homeostasis and detoxification were positively amplified in Brazilian strains. Strains producing bioethanol from corn, on the other hand, must cope with high ethanol concentration and high osmotic pressure, thus gene families related to membrane maintenance were often amplified.

All the strains in the dataset were investigated for CNV by considering S288C as reference. The full list of ORFs showing a CNV for at least one of the strains is reported in Supplementary Table 3. The analysis of over-represented genes was performed for three selected GO terms, namely those of utmost importance for bioethanol production (stress response, energy metabolism and transmembrane transport). The copy number was represented on a heatmap by color scale to better understand the relative abundance among the clusters of strains (Supplementary Table 4). The gene identifiers were pooled together and ordered by the location on the chromosomes, while strains were grouped according to their current industrial application.

Out of 199 genes considered in the heatmap, 130 were related to the transport mechanism. These included transmembrane transporters (73) but also the vesicle trafficking (53) from the endoplasmic reticulum and the Golgi apparatus, then moving to the secretory pathway (toward the plasma membrane) or the vacuole. L20 showed a great number of amplified genes related to the early vesicle-mediated transport (*YPT1*, *SEC23*, *ERP1*, *ERP2*, *ERV46*, *EMP47*, *BST1*, *GYP8*, *RET2*, *MST27*, and *VPS8*) but also exocytosis/secretion (*SYN8*, *SNC1*, *SRO77*, *SEC4*, and *SED4*). Transportation across the cell membrane is ensured by membrane transporters. Such over-represented genes in L20 belong to amino acid (*AVT5*, *AGP1*, *DIP5*, *AGP3*, *GAP1*, *VBA3*, *VBA5*, and *RHB1*), oligopeptide (*OPT2*), acetate (*ADY2*), allantoate (*DAL5*, *DAL4*), carboxylic acid (*JEN1*, *BIO3*, *BIO4*, and *BIO5*), glycerol (*AQY3*), glycerol-3-phosphate (*GIT1*), zinc ion (*ZRT1*), metal (*ALR2*, *ENB1*, *FET5*, and *NFT1*), organic hydroxy compound (*HOL1*), water (*AQY1*), nucleoside (*FUN26*) transporters. The monocarboxylic acids transporter *MCH2* was not amplified in L20 and Ethanol Red but duplicated in other wine and bioethanol strains (CAT-1; Babrzadeh et al., 2012). The allantoate transporter *SEO1* was absent in L20 but duplicated in Ethanol Red and many other strains (CAT-1; Babrzadeh et al., 2012).

In *S. cerevisiae*, sugar transporters play a critical role in biomass utilization by linking the extra- to the intra-cellular compartment. The number of genes involved in monosaccharide transmembrane transport was 12. *MALx*1 are low-affinity sucrose-H + symporters involved in maltose fermentation, which accounts for 50 to 60% of the total fermentable sugars in wort. The CNV analysis reported that Ethanol Red had a large amplification of *MAL31* on chromosome II (5–6 copies), in agreement with the findings from Nagamatsu et al. (2021). With a few exceptions, Bioethanol strains showed at least a duplication of *MAL31*, while L20 was the only wine strain showing a higher copy number (2.5 copies). With regards to sugar metabolism, the L20 strain showed duplication for glucokinase (*GLK1*) and hexokinases (*HXK1* and *HXK2*) involved in the glycolytic process, as well as the alcohol dehydrogenase (*ADH4*) involved in fermentation.

The extracellular environment is continuously changing during fermentation, thus yeast cells are adapting their metabolic response to different conditions (Bauer and Pretorius, 2000). Of the 199 genes, 69 were attributed to the GO term of stress response. Many genes related to the oxidative (*NTG1*, *FRM2*, *HBN1*, *MXR2*, *GRX1*, *HSP30*, *HCM1*, *TRX3 CMK1*, *CUP1-1*, *CUP1-2*, *HYR1*, *MDL2*, *GEX1*, and *GEX2*) and osmotic (*HSP30* and *YPD1*) stress were amplified in the L20 strain. The *HSP30* and *HSP12* genes play a critical role in ethanol-induced stress, protecting the plasma membrane integrity. It is noteworthy to mention that among all the strains considered, L20 was the only strain that had an over-representation of both oxidative and stress genes.

This analysis revealed the genomic peculiarities of the L20 strain when compared to other relevant industrial strains. Moreover, the occurrence of higher copy numbers of genes linked to sugar transport (i.e., *GLK1*, *HXT6*, *HXT7*, *MAL31*, and *MCH2*) and ethanol tolerance (i.e., *HSP12*) support the higher ethanol production performance of strain L20 when compared to Ethanol Red under high-gravity SSF of broken rice (Gronchi et al., 2019). Furthermore, in strain L20 genes related to secretion had higher CNV compared to Ethanol Red (i.e., *BOI1*, *SEC4*, *SNC1*, *SRO77*, *SWH1*, and *SYN8*).

Overall, an important fraction of CNVs is localized in L20 on the chromosomes I, III, and VI, and a distinguishable CNV pattern can be observed for the bioethanol producers. The latter strains share a considerable number of deletions (red boxes in Supplementary Table 4) that are not common in wine and ale/rhum strains, confirming the evolutionary distance reported in Figure 2. Rather than a higher number of gene copies, strain L20 showed an amplification (mostly duplication) of a high number of genes correlated to the selected GO terms. EC1118, CBS7959, CBS7963, SA.9.2.BL3, and RP11.4.14 showed amplification for chromosomes I, III, and VI but none of them showed a similar CNV as was observed for L20.

### Genomic Organization of *Saccharomyces cerevisiae* L20

A total of 5,626 ORFs were predicted for the nuclear genome of L20, out of which 4,903 were shared with S288C. Up to 43 Ty elements were identified in L20 (27 Ty1, 13 Ty2, 2 Ty3, 1 Ty4, and 0 Ty5). The number of delta sequences in L20 was higher than in Ethanol Red (263 versus 237, respectively), whereas 298 are annotated in S288C.

With reference to the genome of S288C, seven specific ORFs were detected in L20 (Supplementary Table 5). The RPS-BLAST annotation showed that such sequences codify for proteins belonging to the amino acid permease (SdaC), mannitol dehydrogenase (Mannitol_dh_C), acetate uptake transporter (Grp1_Fun34_YaaH), and superoxide dismutase (SodA) superfamilies.

### Screening of Recombinant Amylolytic Strains

The L20 and Ethanol Red strains were used as hosts for the expression of *A. tubingensis amyA* and *glaA* genes using delta integration and CRISPR/Cas9 strategies. For delta integration, linear amylase cassettes were constructed to randomly integrate at delta sites in combination with a *kanMX* cassette. The non-site-directed and random nature of delta integration resulted in large phenotypic variability in amylase secretion among isolates. This was evident when recombinants were cultivated on starch-containing plates and evaluated after Lugol staining (data not shown). Those displaying the largest halos were considered as the most efficient amylases secretors and designated as L20 dT8, dT12, dT25, and dT53 (*S. cerevisiae* L20 derivatives), as well as ER dT16, dT17, and dT22 (*S. cerevisiae* Ethanol Red derivatives), and were selected for further strain characterization. For the CRISPR/Cas9 strategy, a three-plasmid system was used to integrate the amylase cassettes into specific target sites in a controlled approach (IS4.1 and/or IS7.1; Claes et al., 2020), and was successfully implemented for both parental strains. Recombinants were designated according to the locus of integration and the amylase sequence.

The mitotic stability of delta or CRISPR/Cas9 recombinants was demonstrated by the preservation of antibiotic resistance and/or hydrolytic activity after 80 generations. PCR was performed to confirm gene integration (data not shown).

### Expression of Heterologous Amylases

**FIGURE 4:**
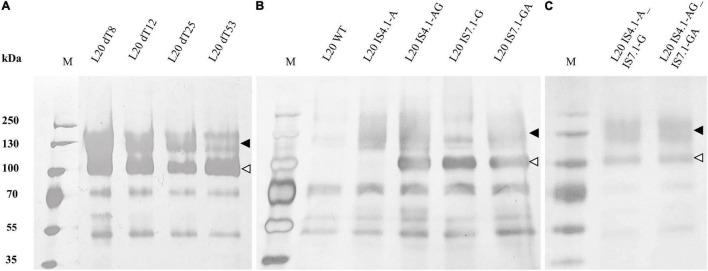
Supernatant from 72-h cultures of *S. cerevisiae* L20 strains was subjected to SDS-PAGE followed by silver staining. Arrows indicate the presence of recombinant protein species (▲) AmyA and (△) GlaA in the supernatant: **(A)** delta integrated, **(B,C)** CRISPR/Cas9 recombinants. WT indicates the parental strain. The PageRuler Prestained Protein Ladder (Fermentas) was used as protein size marker (M).

Recombinant L20 strains were cultured in YPD for 72 h and the supernatant used for SDS-PAGE analysis to confirm the secretion of heterologous amylases (Figure 4).

The SDS-PAGE analysis showed that AmyA and GlaA proteins were produced as differentially glycosylated species, with an average molecular size of 120 and 100 kDa, respectively. Similar results were found for Ethanol Red variants (Supplementary Figure 1). This is in agreement with previous studies (Viktor et al., 2013; Cripwell et al., 2017, 2019a,b).

**FIGURE 5:**
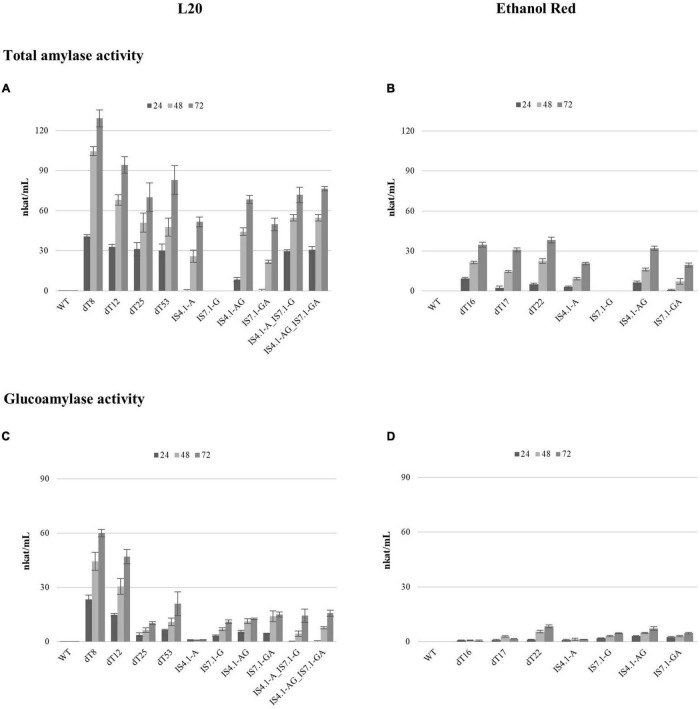
The total amylase **(A,B)** and glucoamylase **(C,D)** activity displayed by the *S. cerevisiae* L20 and Ethanol Red strains expressing *amyA* and/or *glaA* genes from *A. tubingensis*. WT indicates the parental strain. Enzymatic activity was determined using cell-free supernatant from cultures after 24, 48, and 72 h of incubation in YPD broth. Error bars represent the standard deviation from the mean of three replicates.

The extracellular amylase activity was evaluated using liquid assays at 50°C on soluble starch (Figure 5).

The DNS assay for secreted amylases revealed that enzymatic activity increased steadily for all strains over time (Figures 5A,B). However, the delta integrated strains showed a considerably higher hydrolytic activity, compared to those constructed using CRISPR/Cas9. L20 delta recombinants showed an average activity of 94 nkat/mL after 72 h of cultivation, which is 1.5-fold higher than the average activity obtained from the CRISPR/Cas9 recombinants (64 nkat/mL) (Figure 5A). The best performing L20 transformant was dT8, which exhibited 129 nkat/mL after 72 h growth. This varying degree of activity could be explained by the number/location of gene copies that were integrated.

Unexpectedly, such a large activity discrepancy among recombinants was not reported for Ethanol Red. The average activity displayed by delta integrated strains (35 nkat/mL) was 0.25-fold higher than the CRISPR/Cas9 strains (28 nkat/mL). The strain showing the highest activity was ER dT22 (38 nkat/mL at 72 h), which displayed a 1.36-fold higher activity than the CRISPR/Cas9 derivatives.

The activity of the CRISPR/Cas9 recombinant strains was significantly lower compared to those constructed using delta integration, and the results provide some interesting discussion points. Despite the locus, integration of a single, as well as both gene cassettes (simultaneously) resulted in much higher enzymatic activities in the case of L20 variants. By cloning a single *amyA* copy in locus IS4.1 (indicated by IS4.1-A), the L20 recombinant strain reached 51 nkat/mL after 72 h, whereas the maximum activity for an Ethanol Red transformants reached only 21 nkat/mL. When a double cassette *amyA*-*glaA* was inserted in the same locus (indicated by IS4.1-AG), in Ethanol Red, the activity increased by 0.52-fold (32 nkat/mL), while in strain L20 it only improved by 0.33-fold (68 nkat/mL). On average, a 2.1-fold higher activity was displayed for L20 compared to Ethanol Red strains. Moreover, when the same combination was integrated into the IS7.1 locus (strains IS7.1-GA) the hydrolytic activity was only 50 nkat/mL for L20 and 19.5 for Ethanol Red strains. Thus, the L20 strain showed a consistently higher activity over the Ethanol Red strain (2.56-fold).

Noteworthy, the L20 strain with the *amyA* and *glaA* cassettes integrated singularly (L20 IS4.1-A_IS7.1-G) and the transformant with two genes at both loci (L20 IS4.1-AG_IS7.1-GA) showed an activity of 71 and 76 nkat/mL, respectively, whereas a theoretical 2-fold improvement was expected.

This may be explained by the position of the integration event and the possible alteration of the chromosome structure, transcriptome, and epigenome (Flagfeldt et al., 2009; Wu et al., 2017; Gui et al., 2021). Wu et al. (2017) determined in a transcriptomic study the expression of an integrated fluorescent protein gene into different codifying genomic loci in *S. cerevisiae*, which revealed a genomic landscape of position effects besides the telomere and centromere regions. By observing their results, the closest codifying loci to our gRNA targets were considered as moderate (IS4.1) and high expression (IS7.1) levels. In this study, however, the heterologous cassette was inserted in intergenic regions, which are differently regulated and can result in modulated expression (Flagfeldt et al., 2009).

The glucoamylase activity assay revealed the same discrepancy between delta and CRISPR/Cas9 recombinants (Figures 5C,D), in agreement to those reported from the DNS assay (Figures 5A,B). L20 dT8 displayed a glucoamylase activity of 60 nkat/mL after 72 h and the average activity among L20 delta strains was 34.5 nkat/mL (Figure 5C). On the other hand, in L20 recombinants obtained by CRISPR/Cas9 the average was 14 nkat/mL. This could once again be due to the higher gene copy number integrated into the delta recombinants compared to the CRISPR/Cas9 strains. Strains containing a single *glaA* in IS7.1 (namely L20 IS7.1-G, L20 IS7.1-GA and L20 IS4.1-A_IS7.1-G) or the IS4.1 locus (L20 IS4.1-AG) displayed similar activity levels. Unexpectedly, the glucoamylase activity of L20 IS4.1-AG_IS7.1-GA, which contained two *glaA* copies was only slightly higher than L20 IS4.1-AG, which had a single *glaA* integration (16 and 13 nkat/mL, respectively).

Glucoamylase activity is well known to be limited to the availability of starch non-reducing ends (Görgens et al., 2015) produced by α-amylases, and this indicates that an α-amylase:glucoamylase ratio of 1:1 is not optimal for efficient starch hydrolysis. Therefore, higher α-amylase titers are required.

A possible explanation for the comparatively lower enzymatic activity for Ethanol Red derivatives could be a constitutive resilience of Ethanol Red to genome editing (as reported by Zhang et al., 2014 in industrial strain *S. cerevisiae* ATCC 4124), or a lower number of delta sequences compared to strain L20. Furthermore, in the case of the CRISPR/Cas9 engineered strains, Ethanol Red derivatives demonstrated a lower enzymatic activity compared to the respective L20 strains, suggesting that the intraspecific genomic variability plays a fundamental role in gene expression and, therefore, in construction of strains with high performance.

To our knowledge, this is one of the first reports demonstrating this in *S. cerevisiae* engineering for amylase production and will be of great importance to support the future development of efficient amylolytic CBP strains.

### Genome Sequencing of Recombinant Strains

The use of delta sequences as target for genomic integration allows the simultaneous construction of strains with a varying number of gene copies and, therefore, different ratios of amylase:glucoamylase genes (Table 5).

**TABLE 5:** Results of Illumina sequencing of recombinant *S. cerevisiae* L20 and Ethanol Red strains obtained in this study.

*S. cerevisiae*	L20				Ethanol Red		
	dT8	dT12	IS4.1-AG	IS7.1-GA	dT16	IS4.1-AG	IS7.1-GA
Number of paired-end reads (2 × 150 bp)	11,832,827	10,340,011	8,977,407	9,557,402	10,911,300	10,478,341	9,915,227
Number of contigs	188	201	190	193	230	266	243
Genome coverage (x-fold)	98	75	72	87	70	71	69
Average genome size (Mb)	11.6	11.6	11.6	11.6	11.5	11.6	11.6

**Genes for BLAST analysis/Coverage**							

*amyA*	48.4 (***1.02***)	33.5 (***0.96***)	38.0 (***1.11***)	43.7 (***1.01***)	34.9 (***1.08***)	40.8 (***1.21***)	38.2 (***1.20***)
*glaA*	119.8 (***2.51***)	95.3 (***2.73***)	38.8 (***1.13***)	48.7 (***1.13***)	ND	63.4 (***1.88***)	39.1 (***1.23***)
*ACT1*	51.2	37.7	36.3	48.9	33.9	34.3	32.9
*ALG9*	48.8	34.9	34.8	41.9	32.3	36.1	32
*PGK1*	45.3	33.3	32.8	41.3	31.9	31.7	31.5
*TFC1*	45.3	34.2	33.6	40.2	31.5	32.5	30.9
Average coverage of reference genes	47.6	35	34.4	43	32.4	33.7	31.8

*The copy number of the integrated amyA and glaA genes was calculated based on the coverage of reference genes.*

*Bold italic fonts report copy numbers integrated into each genome estimated considering the ratio between the average coverage of the integrated genes and the average coverage of the four reference genes.*

*ND, not detected.*

The copy numbers of *amyA* and *glaA* in recombinant strains were consistent with the methodology used. The highest number of gene copies (1.02 for *amyA* and 2.51 for *glaA*) was found for L20 dT8, in accordance with the results of the enzymatic assays where L20 dT8 showed the highest activity (Figures 5A,C). Considering L20 dT8 as the best amylase producer, it can be assumed that the α-amylase:glucoamylase ratio of 1:2.5 (1.02:2.51) represent the baseline for further increase in gene copies and enzymatic activity. However, the dissimilar activity levels displayed by those having a single gene copy (CRISPR/Cas9 approach), suggested that external factors might affect the gene expression, resulting in lower enzymatic activities. As previously mentioned, the integration events could induce chromosome alterations and alter the transcriptome (Flagfeldt et al., 2009; Wu et al., 2017; Gui et al., 2021).

### Starch Conversion of Engineered L20 Derivatives

**FIGURE 6:**
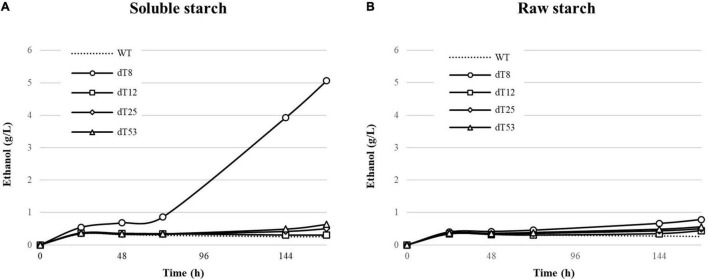
Ethanol production from 2% (w/v) soluble **(A)** and raw starch **(B)** by delta integrated *S. cerevisiae* L20 recombinants. WT indicates the parental strain. Strains were cultivated in YP medium with 0.05% glucose supplementation in oxygen-limited conditions. Values represent the mean of three replicates. The parental strain was used as reference.

The recombinant strains demonstrating the highest enzymatic activity were further examined for their ability to convert soluble and raw starch (2% w/v) to ethanol under CBP conditions and high cell loading (OD_600_ 5; Figure 6).

All strains produced around 0.25 g/L ethanol (Figure 6A) from soluble starch within the first 24 h, corresponding to the theoretical conversion of the glucose supplementation. No further alcohol production was observed after this time point, indicating insufficient starch hydrolysis, except for L20 dT8 which produced up to 4 g/L ethanol (35% of theoretical yield) after 144 h, and this is in accordance with the corresponding enzyme activity (Figure 5A). By contrast, the other L20 recombinants (L20 dT25 and dT53) showed a modest ethanol production after 120 h. In particular, the strain dT12, although displaying good promise in terms of enzymatic activities at 50°C (Figure 5) produced ethanol levels slightly higher than those of the parental. This finding can be explained considering that at 30°C, temperature adopted for the CBP setting, both enzymes sharply decreased their activity (Viktor et al., 2013), thus releasing limited amount of glucose to support yeast cell growth. Moreover, the use of delta integration results in transformants with varying degrees of activity (Romanos et al., 1992; Cho et al., 1999; Favaro et al., 2012, 2015), which might not necessarily correlate to their fermentative abilities. Gene integrations can have caused a metabolic burden on *S. cerevisiae* L20 dT12 which in turn affects the strain’s ability to grow and ferment in a CBP context. This hypothesis is under investigation to further expand the scientific knowledge about metabolic burden in *S. cerevisiae* strains engineered for the expression of heterologous genes (Wu et al., 2016; Favaro et al., 2019a; Zahrl et al., 2019). The ethanol production from soluble starch was consistent with Nakamura et al. (1997) where the glucoamylase producing strain *S. cerevisiae* SR93 reached 3.3 g/L of ethanol after 48 h. Similarly, in Favaro et al. (2012) *S. cerevisiae* F2 and F3 produced 5.4 and 4.8 g/L of ethanol after 48 h, respectively.

On raw corn starch, the average ethanol production after 144 h was 0.48 g/L. As expected, L20 dT8 produced the highest ethanol titers 0.67 g/L (Figure 6B; 6% of theoretical yield). Despite the promising preliminary results, the hydrolytic activity was not sufficient to support the starch-to-ethanol route.

The *amyA* and *glaA* from *A. tubingensis* have previously been expressed using a multi-copy plasmid platform to engineer the *S. cerevisiae* Mnuα strain (Viktor et al., 2013). The recombinant strain was able to reach 80% of theoretical ethanol yield on 2% raw corn starch, demonstrating the hydrolytic ability of the amylases. Therefore, it is hypothesized that an increase in integrated copy number would improve the overall conversion of starch for the Ethanol Red and L20 derivatives. To enhance amylase secretion, further analysis has to be performed to identify the most favorable number of heterologous gene copies, as well as, the best amylase:glucoamylase ratio, while at the same time avoiding phenotypic alteration of the recombinant yeast strains. However, the lack of linearity between the number of integrated gene copies and the enzymatic activity suggests that the expression may be influenced by other, possibly strain-specific factors.

Overall, two different techniques were successfully employed for the development of amylase-producing yeast. They differ in terms of specificity of the target, number of gene copies and outputs. The delta integration approach resulted in recombinant strains displaying variable degrees of activity. The screening of numerous colonies could be time-consuming and difficult to handle. On the other hand, the CRISPR/Cas9 approach allows for a fine selection of target sites and modulation of gene copy numbers.

However, the combination of both approaches may lead to important advancements in CBP strain construction. For evaluation of a large number of recombinants concurrently, delta integration can ensure a rapid sorting of the most efficient in terms of saccharification and ethanol yields. After genome sequencing, the optimal ratio can be customizable and fine-tuned using CRISPR/Cas9.

Although the CRISPR/Cas9 approach was successful, one round of transformation was not sufficient for effective starch hydrolysis. Consistent improvements are expected to be achieved by identifying suitable genomic loci to integrate additional amylase copies (Jessop-Fabre et al., 2016).

In this work, a natural *S. cerevisiae* strain was described as a promising alternative for the development of a future CBP yeast. Genomic insight into L20’s genome revealed a distinctive profile for cellular transport systems, not only in terms of fermentative abilities but also for vesicle trafficking and secretion. This makes *S. cerevisiae* L20 an ideal candidate for the expression of heterologous hydrolase genes, which is fundamental for a CBP configuration. Future studies will investigate the fine tuning of amylase copy number for the efficient saccharification of starch, using L20 (or one of its derivatives).

## Data Availability Statement

The datasets presented in this study can be found in online repositories. The names of the repository/repositories and accession number(s) can be found below: PRJNA762028.

## Author Contributions

NG performed the genome engineering, enzymatic activities, and fermentations experiments, participated in the experimental design, performed data analysis and data interpretation, and drafted the original manuscript. ND and LT performed genome assembly and whole genome analysis. RC participated in CBP strain construction, commented on the manuscript, and funding acquisition. MF-M participated in CBP strain construction. StC, JT, and WV commented and revised the manuscript. MB funding acquisition and commenting the revised manuscript. LF conceptualized the study and the experimental design, supervised the investigation, data interpretation, funding acquisition, editing and revision of the manuscript. SeC funding acquisition and commented the revised manuscript. All authors read and approved the final manuscript.

## Conflict of Interest

The authors declare that the research was conducted in the absence of any commercial or financial relationships that could be construed as a potential conflict of interest.

## Publisher’s Note

All claims expressed in this article are solely those of the authors and do not necessarily represent those of their affiliated organizations, or those of the publisher, the editors and the reviewers. Any product that may be evaluated in this article, or claim that may be made by its manufacturer, is not guaranteed or endorsed by the publisher.

## Footnotes

1
https://itol.embl.de/

## Supplementary Material

Supplementary Figure 1 SDS-PAGE analysis of 72-h culture of recombinant *S. cerevisiae* Ethanol Red strains followed by silver staining. Arrows indicate the presence of recombinant protein species (▲) AmyA and (△) GlaA in the supernatant. WT indicates the parental strain. The PageRuler Prestained Protein Ladder (Fermentas) was used as protein size marker (M).
