# Supplementary figures and images for: Natural Saccharomyces cerevisiae Strain Reveals Peculiar Genomic Traits for Starch-to-Bioethanol Production: the Design of an Amylolytic Consolidated Bioprocessing Yeast
Source: Front Microbiol. 2022 Jan 20;12:768562. doi: 10.3389/fmicb.2021.768562 (PMC8815085; doi:10.3389/fmicb.2021.768562)

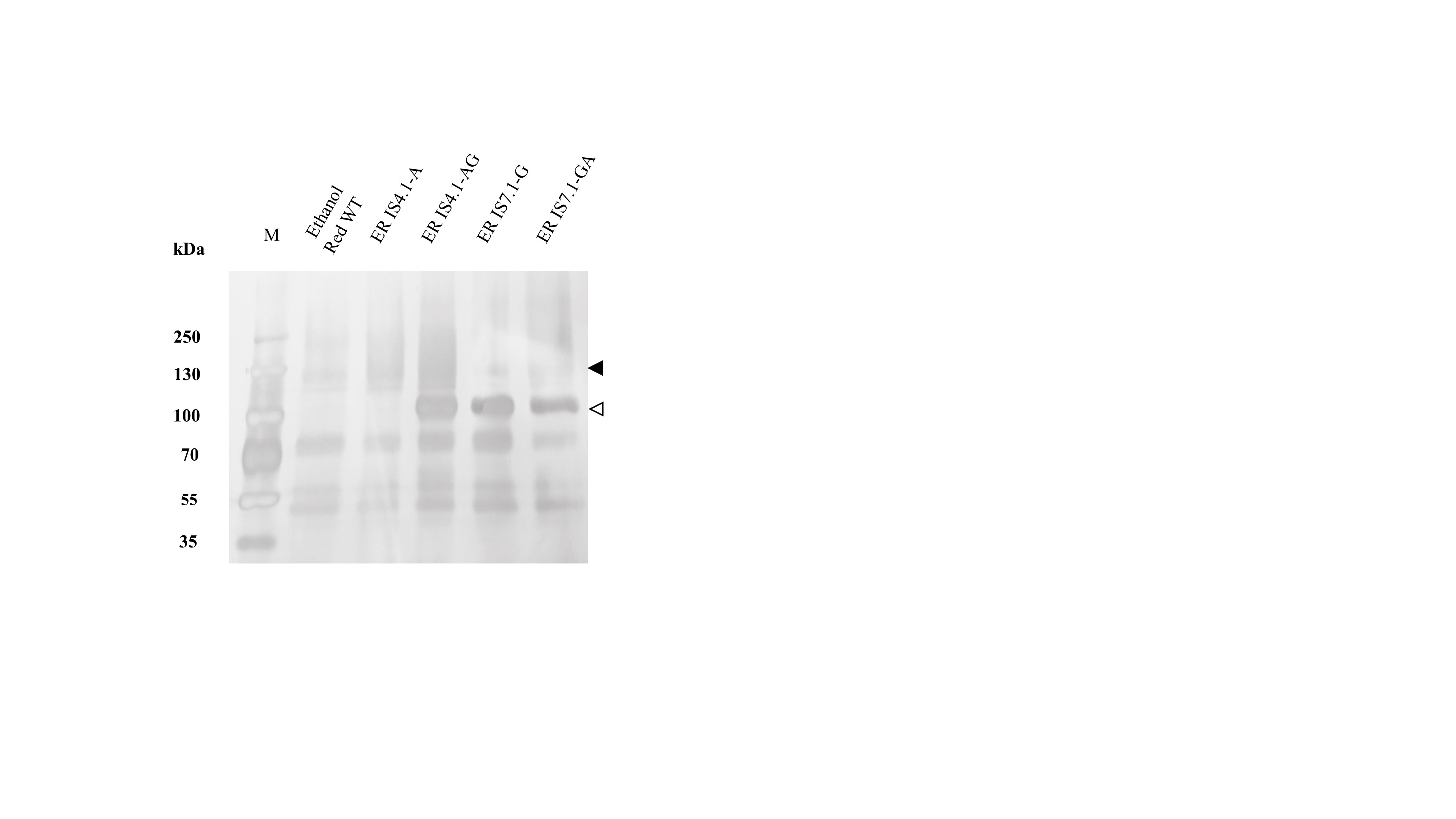

Supplement: Supplementary Figure 1 — SDS-PAGE analysis of 72-h culture of recombinant S. cerevisiae Ethanol Red strains followed by silver staining. Arrows indicate the presence of recombinant protein species (▲) AmyA and (△) GlaA in the supernatant. WT indicates the parental strain. The PageRuler Prestained Protein Ladder (Fermentas) was used as protein size marker (M). [file Image_1.jpg]
